# Exploratory Work in the Quaternary System of Ca–Eu–Cd–Sb: Synthesis, Crystal, and Electronic Structures of New Zintl Solid Solutions

**DOI:** 10.3390/ma11112146

**Published:** 2018-10-31

**Authors:** Alexander Ovchinnikov, Gregory M. Darone, Bayrammurad Saparov, Svilen Bobev

**Affiliations:** 1 Department of Chemistry and Biochemistry, University of Delaware, Newark, DE 19716, USA; alexovc@udel.edu (A.O.); gdarone@udel.edu (G.M.D.); saparov@udel.edu (B.S.); 2 Charter School of Wilmington, Wilmington, DE 19807, USA; 3 Department of Chemistry and Biochemistry, University of Oklahoma, Norman, OK 73019, USA

**Keywords:** Zintl phases, antimonides, crystal structure, chemical bonding

## Abstract

Investigation of the quaternary system, Ca–Eu–Cd–Sb, led to a discovery of the new solid solutions, Ca_1−*x*_Eu*_x_*Cd_2_Sb_2_, with the CaAl_2_Si_2_ structure type (*x* ≈ 0.3–0.9, *hP*5, *P*$\bar{3}$*m*1, *a* = 4.6632(5)–4.6934(3) Å, *c* = 7.630(1)–7.7062(7) Å), Ca_2−*x*_Eu*_x_*CdSb_2_ with the Yb_2_CdSb_2_ type (*x* ≈ 0.6, *oS*20, *Cmc*2_1_, *a* = 4.646(2) Å, *b* = 17.733(7) Å, *c* = 7.283(3) Å), and Eu_11−*x*_Ca*_x_*Cd_6_Sb_12_ with the Sr_11_Cd_6_Sb_12_ type (*x* ≈ 1, *mS*58, *C*2/*m*, *a* = 32.407(4) Å, *b* = 4.7248(5) Å, *c* = 12.377(1) Å, *β* = 109.96(1)°). Systematic crystallographic studies of the Ca_1−*x*_Eu*_x_*Cd_2_Sb_2_ series indicated expansion of the unit cell upon an increase in the Eu content, in accordance with a larger ionic radius of Eu^2+^ vs. Ca^2+^. The Ca_2−*x*_Eu*_x_*CdSb_2_ composition with *x* ≈ 0.6 adopts the non-centrosymmetric space group, *Cmc*2_1_, although the parent ternary phase, Ca_2_CdSb_2_, crystallizes in the centrosymmetric space group, *Pnma*. Two non-equivalent Ca sites in the layered crystal structure of Ca_2−*x*_Eu*_x_*CdSb_2_ get unevenly occupied by Eu, with a preference for the interlayer position, which offers a larger available volume. Similar size-driven preferred occupation is observed in the Eu_11−*x*_Ca*_x_*Cd_6_Sb_12_ solid solution with *x* ≈ 1.

## 1. Introduction

Zintl phases constitute a unique family of intermetallic compounds with polar chemical bonding. Due to a significant electron transfer, formal charges can be assigned to parts of crystal structures in such compounds, considering that all elements in a given composition achieve a stable closed-shell (octet) electron configuration [1]. In Zintl phases, the idealized charges are balanced, which can be understood, yet within an oversimplified picture, as an optimal redistribution of electrons among the constituting elements, leading to fully occupied bonding states and empty antibonding levels. Different patterns of hetero- and homoatomic bonding can be realized in Zintl compounds. Consequently, their crystal structures frequently display a high degree of complexity. Many known intermetallic compounds, especially those with group 15 elements, represent electronically balanced phases crystallizing in structures with huge unit cells and large numbers of atoms per cell [2,3,4,5,6,7]. Group 15 elements are commonly referred to as pnictogens, and their compounds with metals are called pnictides, which is the terminology adopted in this paper.

The energetic separation of the bonding and antibonding states in Zintl phases typically result in semiconducting properties [1,8]. Since electronegativity differences for metals are not high in comparison to metal-nonmetal pairs, the magnitudes of the electronic bandgaps in Zintl compounds are rather small, suggesting the classification of these materials as narrow-gap semiconductors. This makes Zintl phases promising candidates for thermoelectric applications. High electrical conductivity, related to narrow bandgaps, and low thermal conductance, resulting from complex crystal structures, are important prerequisites for high thermoelectric efficiency. In particular, metal pnictides have been actively studied after the discovery of a high thermoelectric figure of merit in Yb_14_MnSb_11_ and related compounds [9,10,11].

Chemical modifications, such as doping, proved to be a convenient tool to optimize electronic properties of Zintl phases. In addition, introduction of extra components can sometimes yield unexpected multinary compounds with new structural features [12,13,14]. In this contribution, we report on the synthesis and the structural characterization of several quaternary phases that were discovered through exploratory work in the system, Ca–Eu–Cd–Sb.

## 2. Materials and Methods

### 2.1. Synthesis

All weighing and loading manipulations were performed inside an Ar-filled glovebox with oxygen and moisture levels of less than 1 ppm. Ca_1−*x*_Eu*_x_*Cd_2_Sb_2_ (*x* ≈ 0.3–0.9) were prepared by the flux technique, using molten lead as a flux. Elemental Ca, Eu, Cd, Sb, and Pb (all with purity >99.9 wt.%) were loaded in alumina crucibles with the molar ratio (Ca + Eu): Cd: Sb: Pb = 1:2:2:10. The crucibles were sealed in evacuated fused silica tubes. After that, the sealed tubes were moved to programmable muffle furnaces, where the reactions were carried out at 1223 K for 24 h (ramp rate 200 K/h). The Pb flux was removed at 773 K (cooling rate of 5 K/h) by centrifugation, after which the tubes were brought back into the glovebox and crack-opened. In all cases, the main products were well-formed single crystals of Ca_1−*x*_Eu*_x_*Cd_2_Sb_2_. However, the refined Ca: Eu ratios were always different in comparison with the nominal compositions. For example, the sample with the starting ratio, Ca:Eu = 1:1, produced Ca_1−*x*_Eu*_x_*Cd_2_Sb_2_ crystals with *x* ≈ 0.7 (Ca:Eu ≈ 1:2.3), whereas the reaction with nominal Ca:Eu = 1:3 yielded crystals with *x* ≈ 0.9 (Ca:Eu ≈ 1:9). In the latter sample, small single crystals of the Ca_2−*x*_Eu*_x_*CdSb_2_ composition with *x* ≈ 0.6 were also found as a side product. Attempts to produce this solid solution in larger quantities starting from the composition, Ca:Eu:Cd:Sb:Pb = 1:1:1:2:10, and using the same temperature profile as before resulted in Eu_11−*x*_Ca*_x_*Cd_6_Sb_12_ with *x* ≈ 1 as the main phase.

### 2.2. Powder X-ray Diffraction (PXRD)

PXRD patterns were collected on a Rigaku Miniflex diffractometer (filtered Cu K*_α_* radiation, *λ* = 1.5418 Å). Data were gathered in a *Θ*–*Θ* mode between 10° and 75° with a step size of 0.05° and 2 s/step counting time. The diffractometer was operated inside a nitrogen-filled glovebox to prevent possible deterioration of the samples upon exposure to air.

A representative PXRD pattern for a Ca_1−*x*_Eu*_x_*Cd_2_Sb_2_ sample is shown in Figure S1 in the Supporting Information. While the Ca_1−*x*_Eu*_x_*Cd_2_Sb_2_ series appear to be air-stable according to the PXRD results, Ca_2−*x*_Eu*_x_*CdSb_2_ and Eu_11−*x*_Ca*_x_*Cd_6_Sb_12_ slowly decompose when exposed to the ambient atmosphere.

### 2.3. Single-Crystal X-ray Diffraction (SCXRD)

Suitable single crystals were picked under dry Paratone-N oil and mounted on low-background plastic loops. Data collection was performed on a Bruker SMART APEX CCD diffractometer equipped with monochromated Mo K*_α_* radiation (*λ* = 0.71073 Å). Data were gathered at 200 K, maintained by a stream of cold nitrogen gas. The raw data were integrated using the program, SAINT [15]. Semiempirical absorption corrections were applied with the SADABS software [16]. Crystal structures were solved by direct methods and refined by full matrix least-squares methods on *F*^2^ using SHELXL [17]. Atomic coordinated were standardized with the program, STRUCTURE TIDY [18]. Full details of the data collection, atomic coordinates, and selected interatomic distances are given in Table 1, Table 2, Table 3, Table 4, Table 5, Table 6, Table 7 and Table 8 and in the Supporting Information. CSD 1873642, 1873643, 1873644, 1873645, 1873646, 1873647, 1873648, and 1875197 contain the supplementary crystallographic data for this paper. These deposited data can be obtained free of charge via http://www.ccdc.cam.ac.uk/conts/retrieving.html (or from the CCDC, 12 Union Road, Cambridge CB2 1EZ, UK; Fax: +44-1223-336033; E-mail: deposit@ccdc.cam.ac.uk).

### 2.4. First-Principle Calculations

Electronic structure calculations were done on the scalar relativistic level for the idealized composition, CaEuCdSb_2_, (a hypothetical member of the Ca_2−*x*_Eu*_x_*CdSb_2_ solid solution with *x* = 1, space group *Cmc*2_1_) using the SIESTA code [19]. The initial atomic coordinates and lattice parameters were taken from the refined Ca_2−*x*_Eu*_x_*CdSb_2_ structure with *x* ≈ 0.6. The AE1 and AE2 sites were treated as fully occupied by Eu and Ca, respectively (see the Results and Discussion for details of the crystal structure). The Perdew-Burke-Ernzerhof parametrization of the generalized gradient approximation (GGA) functional was employed [20]. At first, suitable Troullier-Martins pseudopotentials were generated with the ATOM program and carefully tested on several known compounds to achieve good reproducibility of electronic structures and optimized lattice parameters [21]. After that, atomic coordinates and lattice constants of Ca_2−*x*_Eu*_x_*CdSb_2_ were relaxed down to residual forces of less than 10^−2^ eV/Å. Double zeta polarized orbitals were employed as a basis set. The Brillouin zone was sampled by a 18 × 12 × 10 *k*-point grid and the mesh cut-off was set to 800 Ry after checking for convergence. Strong correlations of the Eu 4f orbitals considered by applying the GGA + *U* approach with *U*_eff_ = 7 eV (rotationally invariant formulation [22]). For consistency with the previous theoretical work on d^10^ metal antimonides [23,24], we also introduced *U*_eff_ = 2 eV for the Cd 4d states. Spin polarization was treated in a simple ferromagnetic model. The forces were relaxed down to less than 10^−2^ eV/Å. Chemical bonding investigation was performed using the Crystal Orbital Hamilton Population analysis [25] as implemented in SIESTA.

## 3. Results and Discussion

### 3.1. Crystal Structure of the Ca_1–x_Eu_x_Cd_2_Sb_2_ Solid Solution

**Figure 1:**
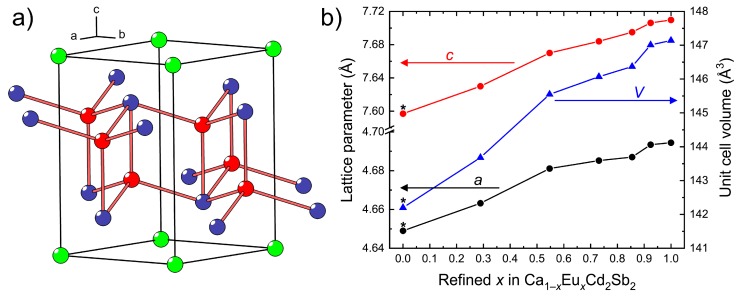
(**a**) Crystal structure of the CaAl_2_Si_2_-type Ca_1−*x*_Eu*_x_*Cd_2_Sb_2_ solid solution. The Ca/Eu, Cd, and Sb atoms are shown in green, red, and blue, respectively; (**b**) variation of the lattice parameters and unit cell volume of Ca_1−*x*_Eu*_x_*Cd_2_Sb_2_ as a function of the Eu content. The lattice parameters of the end members, i.e., the ternary phases CaCd_2_Sb_2_ and EuCd_2_Sb_2_, are included in the plot, but comparing the metrics, it must be born in mind that the literature data for CaCd_2_Sb_2_ (marked with asterisks) are from room temperature [26] and that the sample is prepared via a different synthetic route. The parameters for EuCd_2_Sb_2_ are from own data (CSD 1875197), whereby the crystal has been grown the same way as Ca_1−*x*_Eu*_x_*Cd_2_Sb_2_.

The Ca_1−*x*_Eu*_x_*Cd_2_Sb_2_ solid solution crystallizes in the space group, *P*$\bar{3}$*m*1, and adopts the CaAl_2_Si_2_ structure type (Pearson code *hP*5), which can be also viewed as a ternary derivative of the La_2_O_3_ type (Figure 1a). Atomic coordinates and equivalent displacement parameters for Ca_1−*x*_Eu*_x_*Cd_2_Sb_2_ with *x* = 0.29(1) are presented in Table 2. Crystallographic data for the other studied compositions are given in the Supporting Information.

The crystal structure accommodates two-dimensional slabs of corner- and edge-sharing CdSb_4_ tetrahedra alternating with hexagonal layers of *AE* atoms, where *AE* = Ca + Eu. The *AE* atoms are six-fold coordinated by the adjacent Sb atoms, forming almost perfectly regular octahedra. Assignment of the formal charges to the atoms in the structure yields an electron-balanced composition according to the notation (*AE*^2+^)(Cd^2+^)_2_(Sb^3−^)_2_. The end members of these series with *x* = 0 (CaCd_2_Sb_2_) and *x* = 1 (EuCd_2_Sb_2_) are also known and crystallize in the same structure [26]. In addition, various other pnictides with the *AETM*_2_*Pn*_2_ composition (*AE* = alkaline-earth metal, Eu, or Yb; *TM* = transition metal; *Pn* = pnictogen) adopt this type [27,28,29]. Many of these phases have been studied as potential thermoelectric materials owing to their narrow-gap semiconducting properties [30,31,32,33,34,35,36].

In the Ca_1−*x*_Eu*_x_*Cd_2_Sb_2_ series, the lattice parameters and the unit cell volume gradually increase on going from the Eu-poorer to the Eu-richer compositions (Figure 1b), in accordance with a larger ionic size of Eu^2+^ in comparison with Ca^2+^ (for the same coordination number 6 for instance, *r*(Ca^2+^) = 1.00 Å, *r*(Eu^2+^) = 1.17 Å [37]). The Vegard’s law is not strictly observed, i.e., the change of the unit cell dimensions is not linear, suggesting that local ordering may be taking place. Analysis of the single-crystal diffraction data gives no indication of superstructure reflections, thus confirming the preservation of the long-range CaAl_2_Si_2_ structure.

In line with the increasing average size of the *AE*^2+^ cation in *AE*Cd_2_Sb_2_ (*AE* = Ca + Eu), the *AE*–Sb contacts become progressively longer upon increasing the level of Eu substitution (Table 3). At the same time, the Cd–Sb bonds remain virtually unaffected. Another noteworthy structural change occurring as the Eu concentration increases concerns the Sb–*AE*–Sb bond angles, and the trend is suggestive of adopting a more regular octahedral environment of the *AE* atoms.

### 3.2. Crystal and Electronic Structure of the Ca_2−x_Eu_x_CdSb_2_ Solid Solution (x ≈ 0.6)

A crystal of the new solid solution with the composition, Ca_2−*x*_Eu*_x_*CdSb_2_ (*x* ≈ 0.6), was discovered as a by-product in the sample containing Ca_1−*x*_Eu*_x_*Cd_2_Sb_2_ as the main phase. The crystal structure of Ca_2−*x*_Eu*_x_*CdSb_2_ belongs to the Yb_2_CdSb_2_ type (Pearson code *oS*20, space group *Cmc*2_1_), in contrast to the parent ternary phase, Ca_2_CdSb_2_, crystallizing in its own type (Pearson code *oP*20, space group *Pnma*) [38]. The two prototypic structures possess no homoatomic Sb–Sb bonds and can therefore be rationalized within the Zintl concept as (*AE*^2+^)_2_(Cd^2+^)(Sb^3−^)_2_, where *AE* stands for Yb or Ca. The main difference between the two types stems from non-identical stacking of the layers built up of corner-sharing CdSb_4_ tetrahedra. Whereas in the non-centrosymmetric Yb_2_CdSb_2_, the adjacent Cd-Sb layers are related only by translation, in Ca_2_CdSb_2_, similar layers alternate along the stacking direction with their crystallographically inverted counterparts. 

**Figure 2:**
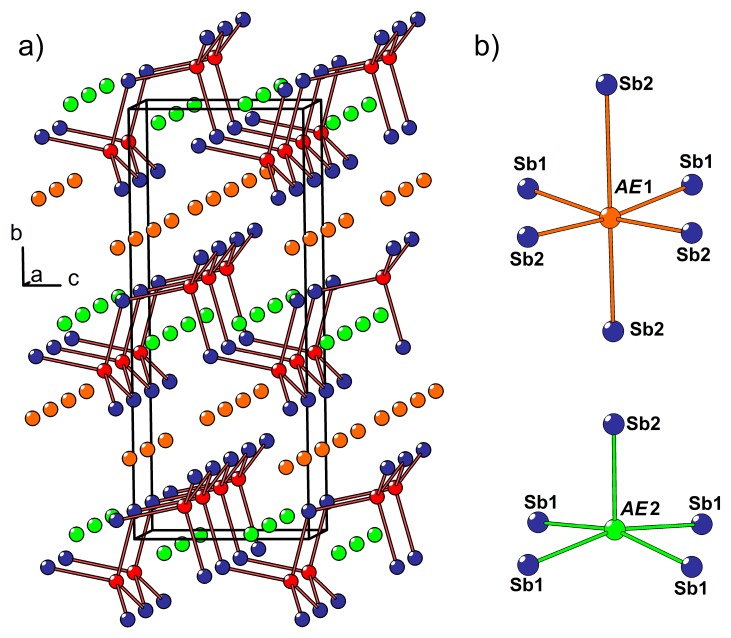
(**a**) Crystal structure of the Ca_2−*x*_Eu*_x_*CdSb_2_ solid solution. The *AE*1, *AE*2, Cd, and Sb atoms are shown in orange, green, red, and blue, respectively; (**b**) coordination environment of the *AE*1 and *AE*2 atoms.

From another point of view, the structures of Yb_2_CdSb_2_ and Ca_2_CdSb_2_ can be understood as two different ways of occupying tetrahedral voids in otherwise isostructural Yb–Sb or Ca–Sb substructures [38]. As local atomic environments in both types appear to be almost identical, subtle electronic or size differences must be responsible for stabilization of one or another structure. As a matter of fact, several pnictides with the “2-1-2” composition have been found to crystallize in the Yb_2_CdSb_2_ structure [39,40], whereas Ca_2_CdSb_2_ remains the only representative of its type. In this regard, it is important to mention that the Zn-variants with the same composition are totally different (defect ZrBeSi-type structure) [41], and the Mn-based “2-1-2” analogs adopt their own structure [42,43]. Within this context, it also is instructive to recall that a ternary compound, “Eu_2_CdSb_2_”, with either a Ca_2_CdSb_2_ or Yb_2_CdSb_2_ structure type, is not known to exist (to date). Apparently, the Ca_2_CdSb_2_ structure becomes destabilized upon isovalent substitution of Ca with Eu, yielding a solid solution with the non-centrosymmetric Yb_2_CdSb_2_ atomic arrangement (Figure 2a). Further optimization of the synthetic procedure will be required to systematically study the structural evolution of the “2-1-2” phase in a wider compositional range and to find the critical Eu content necessary to disrupt the original Ca_2_CdSb_2_ structure.

In the crystal structure of Ca_2−*x*_Eu*_x_*CdSb_2_ (*x* ≈ 0.6), two nonequivalent *AE* sites (*AE* = Ca + Eu) are located between the Cd-Sb layers (*AE*1) and within the layers (*AE*2), respectively. The former site adopts a distorted octahedral coordination by the Sb atoms, while the latter position displays a square-pyramidal coordination (Figure 2b). Due to the substantially different volumes of these two coordination polyhedra, pronounced preferred occupation is observed. The *AE*1 site, offering a larger available volume, accumulates most of the Eu in the structure, whereas the smaller *AE*2 site shows a minute Eu occupancy of about 2%, in line with a larger ionic radius of Eu^2+^ versus Ca^2+^. A similar trend was previously observed for the Yb_2_CdSb_2_-type solid solutions, Sr_2−*x*_*A_x_*CdSb_2_ (*A* = Ca, Yb), Ba_2−*x*_*A_x_*CdSb_2_ (*A* = Ca, Sr, Eu, Yb) [39], and Yb_2−*x*_Eu*_x_*CdSb_2_ [24,39].

**Figure 3:**
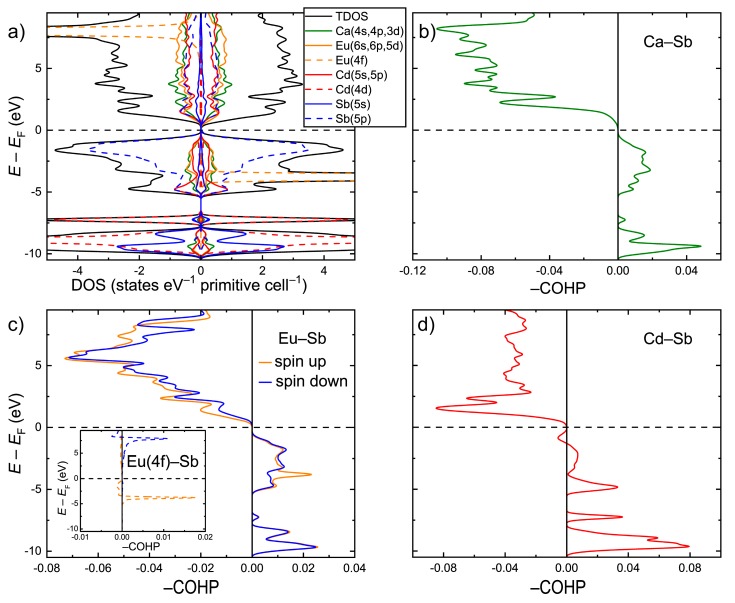
(**a**) Total and projected electronic densities of states (DOS) for the idealized CaEuCdSb_2_ composition; (**b**–**d**) Crystal Orbital Hamilton Population curves (COHP) averaged over selected bonds from Table 6. The inset in (**c**) shows the orbital resolved Eu(4f)–Sb interaction.

Electronic structure calculations were performed within the GGA+*U* scheme for the idealized CaEuCdSb_2_ composition after optimizing the lattice parameters and atomic coordinates. The electronic density of states (DOS) is shown in Figure 3a. In accordance with the simple electron counting in the frame of the Zintl concept, CaEuCdSb_2_ displays an electronic bandgap of about 0.5 eV. This value is larger than the 0.1 eV bandgap calculated for the isostructural YbEuCdSb_2_ [24]. This is likely is an electronic effect due to electronegativity differences between Yb and Ca, suggesting lower iconicity of the bonding in the latter compound. The states just below the Fermi level (*E*_F_) mainly show the Sb(5p) character, indicating a significant electron transfer onto the Sb atoms. A strong hybridization of these states with Cd(5s) and Cd(5p) occurs in the energy window, −5.5 eV < *E*–*E*_F_ < 0. The s, p, and d states of Ca and Eu also demonstrate a sizeable contribution in this energy region. A high peak of the Eu(4f) character is localized around *E*–*E*_F_ = −3.7 eV. The converged magnetic moment on the Eu atom is 7.0 µ_B_, in accordance with the 4f^7^ ground term of the Eu^2+^ ion. Similar to the case of YbEuCdSb_2_, the Eu f orbitals cannot be considered completely inert in this case, since an apparent hybridization with the Sb(5p) states takes place around the peak position. In addition, a “tail” of the 4f peak extends up to the top of the valence band, hybridizing further with the Sb states. Well below the Fermi level, the DOS is dominated by the Sb(5s) states, corresponding to the Sb lone pairs, and by the occupied Cd(4d) states.

Analysis of the chemical bonding with the aid of the Crystal Orbital Hamilton Population curves (COHP) revealed optimized bonding interactions for the Ca–Sb and Eu–Sb pairs (Figure 3c,d). Most importantly, orbital-resolved COHP analysis showed a small, yet non-negligible contribution of the Eu(4f)–Sb orbital mixing to the overall Eu–Sb bonding. The hybridization of the Eu(4f) and Sb(5p) states around the center of the Eu(4f) peak results in a localized set of bonding states, whereas the hybridization just under *E*_F_ gives rise to a broad domain of antibonding character. This finding suggests that the typically core-level 4f orbitals may play a role in the crystal structure formation/selection. The involvement of the 4f states in chemical bonding can be important, alongside atomic size factors, for understanding the stability of compounds that are known to form only with certain rare-earth elements, such as the binary *RE*_3_Bi_7_ phases with *RE* = Nd and Sm [44]. The Cd–Sb contacts display predominantly bonding interactions below *E*_F_, with a small domain of antibonding states in the near vicinity of the Fermi level (Figure 3d). A similar bonding pattern was observed for the Cd–Sb interactions in the prototypic Yb_2_CdSb_2_ structure [38].

### 3.3. Crystal Structure of the Eu_11−x_Ca_x_Cd_6_Sb_12_ Solid Solution (x ≈ 1)

**Figure 4:**
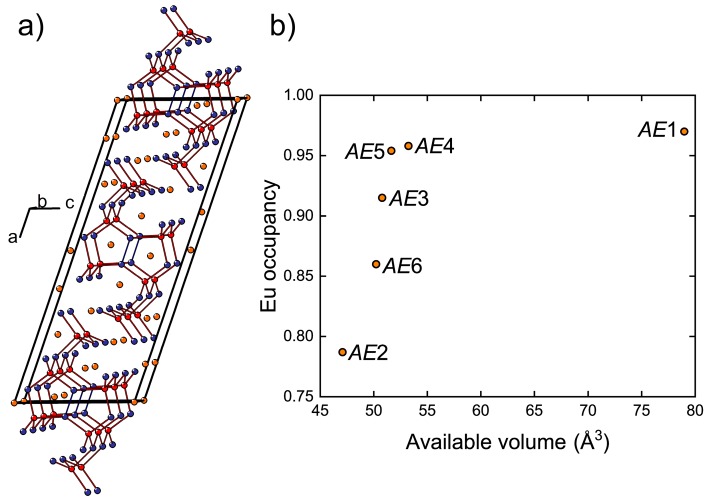
(**a**) Crystal structure of Eu_11−*x*_Ca*_x_*Cd_6_Sb_12_ with Eu/Ca, Cd, and Sb atoms shown in orange, red, and blue, respectively; (**b**) variation of the Eu occupancy as a function of the available volume for the non-equivalent *AE* positions in Eu_10.01_Ca_0.99(1)_Cd_6_Sb_12_ (*AE* = Eu + Ca).

Eu_11−*x*_Ca*_x_*Cd_6_Sb_12_ crystallizes isotypically to the parent Eu_11_Cd_6_Sb_12_ compound (Sr_11_Cd_6_Sb_12_ type, Pearson code *mS*58, space group *C*2/*m* (No. 12), Figure 4a) [45,46]. The refined *x* ≈ 1 in the studied crystal must be close to the upper limit of the Ca substitution for Eu, since the starting mixture contained a large surplus of Ca (recall that the nominal Eu: Ca ratio was 1:1). The crystal structure likely becomes unstable at higher Ca contents due to geometric/size factors. Accordingly, the pure ternary Ca phase has not been reported. This result is in line with the study on the isostructural solid solution, Eu_11−*x*_Yb*_x_*Cd_6_Sb_12_, where the Sr_11_Cd_6_Sb_12_ type is retained only up to *x* ≈ 2 [47]. The similarity between the two quaternary systems is likely related to the close ionic radii of Ca^2+^ and Yb^2+^: *r*(Ca^2+^) = 1.00 Å, *r*(Yb^2+^) = 1.02 Å for coordination number 6 [37].

The crystal structure of Eu_11_Cd_6_Sb_12_ and isostructural compositions has been described in detail previously [45,46,47,48,49]. The anionic substructure contains infinite fragments of corner sharing CdSb_4_ tetrahedra propagating along the monoclinic *b* axis (Figure 4a). Homoatomic Sb–Sb bonding develops within these fragments, resulting in Sb_2_ dumbbells shared between adjacent Cd atoms. In Eu_11−*x*_Ca*_x_*Cd_6_Sb_12_, the *AE* atoms (*AE* = Eu + Ca), except *AE*5, are six-fold coordinated by Sb. *AE*5 resides in a five-fold coordination environment with an average *AE*–Sb bond length of 3.35 Å. A six-fold coordination of *AE*5 can be completed by including an Sb atom located at a much longer distance of 3.98 Å. Similarly, the six-fold coordination of the *AE*1 site (<*d*(*AE*–Sb)> = 3.43 Å) can be regarded as a highly distorted square antiprism (coordination number 8) with two additional Sb atoms at 4.02 Å and 4.03 Å from *AE*1. Although such long distances can hardly be considered bonding interactions, when considered, they provide an estimate of the available volume offered by the Sb coordination environment around each *AE* position. In fact, the occupancy of the *AE* sites by Eu correlates well with the volume of such “extended” polyhedra (Figure 4b). The largest *AE*1 site is occupied mainly by Eu, whereas the highest Ca occupancy is found for the most spatially confined *AE*2 position.

## 4. Conclusions

In summary, using a Pb flux synthesis, we have discovered three new series of solid solutions in the Ca–Eu–Cd–Sb system. The Ca_1−*x*_Eu*_x_*Cd_2_Sb_2_ series extend the field of quaternary compositions crystallizing in the CaAl_2_Si_2_ type. In contrast to these mixed crystals, where the ternary end members adopt the same crystal structure as their solid solution, Ca_2−*x*_Eu*_x_*CdSb_2_ with *x* ≈ 0.6 crystallizes in the Yb_2_CdSb_2_ structure type, whereas Ca_2_CdSb_2_ represents its own type and the hypothetical end member “Eu_2_CdSb_2_” has not been reported. The disruption of the parent Ca_2_CdSb_2_ structure upon Eu doping may be a result of size and electronic factors. Interestingly, our first-principle calculations on the idealized CaEuCdSb_2_ structure indicate that the Eu(4f) states may be relevant for chemical bonding in this phase. Finally, the Eu_11−*x*_Ca*_x_*Cd_6_Sb_12_ solid solution adopts the same structure as the parent Eu_11_Cd_6_Sb_12_ compound (Sr_11_Cd_6_Sb_12_ type). The distribution of Eu over the non-equivalent *AE* sites (*AE* = Eu + Ca) correlates well with the available volume offered by these positions. Although the pure calcium counterpart does not exist, the Eu_11−*x*_Ca*_x_*Cd_6_Sb_12_ appears to extend up to *x* ≈ 1. In the realm of Zintl chemistry, formation of mixed crystals has been widely employed as a tool for electronic property optimization. Results of the present study demonstrate that, in addition to this capability, solid solutions may provide a way of stabilizing crystal structures not observed for the end members of the corresponding series.

## Figures and Tables

**Table 1:** Data collection details and selected crystallographic data for Ca_1−*x*_Eu*_x_*Cd_2_Sb_2_ (space group *P*$\bar{3}$*m*1 (No. 164), *Z* = 1, *T* = 200 K, Mo K*_α_ λ* = 0.71073 Å). The estimated standard deviations on the refined compositions are ±0.01 or better.

Composition	Ca_0.71_Eu_0.29_Cd_2_Sb_2_	Ca_0.45_Eu_0.55_Cd_2_Sb_2_	Ca_0.27_Eu_0.73_Cd_2_Sb_2_	Ca_0.15_Eu_0.85_Cd_2_Sb_2_	Ca_0.08_Eu_0.92_Cd_2_Sb_2_
Formula weight/g mol^−1^	540.83	569.91	590.05	603.48	611.31
*a*/Å	4.6632(5)	4.6811(4)	4.6852(4)	4.687(1)	4.6934(3)
c/Å	7.630(1)	7.670(1)	7.684(1)	7.695(3)	7.7062(7)
*V*/Å^3^	143.68(4)	145.55(3)	146.07(3)	146.37(8)	147.01(2)
*ρ*_calc_/g cm^−3^	6.25	6.50	6.71	6.85	6.90
*µ*_MoKα_/cm^−1^	201.7	224.3	240.9	252.0	257.6
R_1_ [*I* > 2*σ*(*I*)]^a^	0.016	0.012	0.019	0.021	0.016
*w*R_2_ [*I* > 2*σ*(*I*)]^a^	0.031	0.026	0.039	0.038	0.029
R_1_ [all data]^a^	0.018	0.013	0.020	0.023	0.017
*w*R_2_ [all data]^a^	0.031	0.027	0.040	0.038	0.029
*Δρ*_max,min_/e Å^−3^	0.95, −0.96	0.87, −0.79	0.97, −1.08	1.63, −1.01	0.89, −1.27

^a^ R1 = ∑||*F*_o_| − |F_c_||/∑|*F*_o_|; *w*R_2_ = [∑[w(*F*_o_^2^ − *F*_c_^2^)^2^]/∑[w(*F*_o_^2^)^2^]]^1/2^, where w = 1/[*σ*^2^*F*_o_^2^ + (*AP*)^2^ + (*BP*)], and *P* = (*F*_o_^2^ + 2*F*_c_^2^)/3; *A, B* are the respective weight coefficients.

**Table 2:** Atomic coordinates and equivalent displacement parameters (*U*_eq_^a^) for Ca_1−*x*_Eu*_x_*Cd_2_Sb_2_, *x* = 0.29(1).

Atom	Site	*x*	*y*	*z*	*U*_eq_/Å^2^
Ca/Eu^b^	1*a*	0	0	0	0.0125(4)
Cd	2*d*	1/3	2/3	0.36980(7)	0.0157(2)
Sb	2*d*	1/3	2/3	0.75897(6)	0.0128(2)

^a^*U*_eq_ is defined as one third of the trace of the orthogonalized *U*_ij_ tensor. ^b^ 0.711Ca + 0.289(3) Eu.

**Table 3:** Selected bond lengths and angles for Ca_1−*x*_Eu*_x_*Cd_2_Sb_2_ with varied *x*.

Atoms	*x* = 0.289(3)	*x* = 0.547(4)	*x* = 0.731(4)	*x* = 0.855(5)	*x* = 0.924(4)
Ca/Eu–Sb × 6	3.2604(4) Å	3.2847(3) Å	3.2956(4) Å	3.3029(7) Å	3.3097(3) Å
Cd–Sb × 3	2.8660(4) Å	2.8694(3) Å	2.8668(4) Å	2.8646(7) Å	2.8669(3) Å
Cd–Sb	2.9692(9) Å	2.9721(7) Å	2.9692(9) Å	2.966(1) Å	2.9695(8) Å
Sb–Ca/Eu–Sb	88.69(1)°	89.11(1)°	89.40(1)°	89.62(2)°	89.69(1)°
Sb–Ca/Eu–Sb	91.31(1)°	90.89(1)°	90.61(1)°	90.38(2)°	90.31(1)°
Sb–Ca/Eu–Sb	180°	180°	180°	180°	180°
Sb–Cd–Sb	108.89(1)°	109.31(1)°	109.34(2)°	109.17(2)°	109.06(2)°
Sb–Cd–Sb	110.05(1)°	109.63(1)°	109.60(2)°	109.77(2)°	109.88(2)°

**Table 4:** Data collection details and selected crystallographic data for Ca_1.37_Eu_0.63(1)_CdSb_2_ and Eu_10.01_Ca_0.99(1)_Cd_6_Sb_12_ (*T* = 200 K, Mo K*_α_* radiation with *λ* = 0.71073 Å).

Composition	Ca_1.37_Eu_0.63(1)_CdSb_2_	Eu_10.01_Ca_0.99(1)_Cd_6_Sb_12_
Formula weight/g mol^−1^	506.26	3696.76
Space group	*Cmc*2_1_ (No. 36)	*C*2/*m* (No. 12)
*Z*	4	2
*a*/Å	4.646(2)	32.407(4)
*b*/Å	17.733(7)	4.7248(5)
*c*/Å	7.283(3)	12.377(1)
*β*/°	-	109.963(1)
*V*/Å^3^	600.0(4)	1781.2(3)
*ρ*_calc_/g cm^−3^	5.60	6.89
*µ*_MoKα_/cm^−1^	199.0	298.7
R_1_ [*I* > 2*σ*(*I*)] ^a^	0.038	0.028
*w*R_2_ [*I* > 2*σ*(*I*)] ^a^	0.093	0.051
R_1_ [all data] ^a^	0.044	0.037
*w*R_2_ [all data] ^a^	0.096	0.054
*Δρ*_max,min_/e Å^−3^	1.52, −2.57	1.47, −2.02

^a^ R1 = ∑||*F*_o_| − |F_c_||/∑|*F*_o_|; *w*R_2_ = [∑[w(*F*_o_^2^ − *F*_c_^2^)^2^]/∑[w(*F*_o_^2^)^2^]]^1/2^, where w = 1/[σ^2^*F*_o_^2^ + (*AP*)^2^ + (*BP*)], and *P* = (*F*_o_^2^+2*F*_c_^2^)/3; *A*, *B* are the respective weight coefficients.

**Table 5:** Atomic coordinates and equivalent displacement parameters (*U*_eq_^a^) for Ca_2−*x*_Eu*_x_*CdSb_2_, *x* = 0.63(1).

Atom	Site	*x*	*y*	*z*	*U*_eq_/Å^2^
Ca/Eu1 ^b^	4*a*	0	0.3019(1)	0.5306(4)	0.017(1)
Ca/Eu2 ^b^	4*a*	0	0.4782(4)	0.2217(8)	0.013(2)
Cd1	4*a*	0	0.0982(1)	0.3935(4)	0.014(1)
Sb1	4*a*	0	0.0650(1)	0.0026(3)	0.010(1)
Sb2	4*a*	0	0.3243(1)	0.0074(4)	0.014(1)

^a^*U*_eq_ is defined as one third of the trace of the orthogonalized *U*_ij_ tensor. ^b^ Ca/Eu1 = 0.39Ca + 0.61(1)Eu; Ca/Eu2 = 0.98Ca + 0.02(1)Eu.

**Table 6:** Selected interatomic distances for Ca_2−*x*_Eu*_x_*CdSb_2_, *x* = 0.63(1).

Atom Pair	Distance/Å
Ca/Eu1–Sb2 × 2	3.229(2)
Ca/Eu1–Sb1 × 2	3.319(2)
Ca/Eu1–Sb2	3.495(4)
Ca/Eu1–Sb2	3.831(4)
Ca/Eu2–Sb2	3.144(7)
Ca/Eu2–Sb1 × 2	3.188(5)
Ca/Eu2–Sb1 × 2	3.211(5)
Cd1–Sb2 × 2	2.823(2)
Cd1–Sb1	2.908(4)
Cd1–Sb1	3.001(3)

**Table 7:** Atomic coordinates and equivalent displacement parameters (*U*_eq_^a^) for Eu_11–*x*_Ca*_x_*Cd_6_Sb_12_, *x* = 0.99(1).

Atom	Site	*x*	*y*	*z*	*U*_eq_/Å^2^
Eu/Ca1 ^b^	4*i*	0.01907(2)	0	0.68106(6)	0.0104(2)
Eu/Ca2 ^b^	4*i*	0.11381(3)	0	0.50682(7)	0.0096(3)
Eu/Ca3 ^b^	4*i*	0.12576(2)	0	0.01564(6)	0.0105(2)
Eu/Ca4 ^b^	4*i*	0.19980(2)	0	0.34239(6)	0.0099(2)
Eu/Ca5 ^b^	4*i*	0.27714(2)	0	0.13201(6)	0.0106(2)
Eu/Ca6 ^b^	2*a*	0	0	0	0.0095(4)
Cd1	4*i*	0.21983(3)	0	0.66546(9)	0.0109(2)
Cd2	4*i*	0.39866(3)	0	0.25337(8)	0.0102(2)
Cd3	4*i*	0.54755(3)	0	0.24669(9)	0.0126(2)
Sb1	4*i*	0.08999(3)	0	0.23366(8)	0.0100(2)
Sb2	4*i*	0.15114(3)	0	0.78495(8)	0.0097(2)
Sb3	4*i*	0.30966(3)	0	0.47226(8)	0.0096(2)
Sb4	4*i*	0.45328(3)	0	0.11364(8)	0.0092(2)
Sb5	4*i*	0.45647(3)	0	0.49911(8)	0.0093(2)
Sb6	4*i*	0.70360(3)	0	0.13391(8)	0.0091(2)

^a^*U*_eq_ is defined as one third of the trace of the orthogonalized *U*_ij_ tensor. ^b^ Eu/Ca1 = 0.986Eu + 0.014(4)Ca; Eu/Ca2 = 0.788Eu + 0.212(4)Ca; Eu/Ca3 = 0.914Eu + 0.086(4)Ca; Eu/Ca4 = 0.945Eu + 0.055(4)Ca; Eu/Ca5 = 0.938Eu + 0.062(4)Ca; Eu/Ca6 = 0.873Eu + 0.127(5)Ca.

**Table 8:** Selected interatomic distances for Eu_11−*x*_Ca*_x_*Cd_6_Sb_12_, *x* = 0.99(1).

Atom Pair	Distance/Å	Atom Pair	Distance/Å
Eu/Ca1–Sb4 × 2	3.3594(8)	Eu/Ca5–Sb2 × 2	3.2262(8)
Eu/Ca1–Sb5 × 2	3.4130(8)	Eu/Ca5–Sb6 × 2	3.3615(8)
Eu/Ca1–Sb5 × 2	3.5198(9)	Eu/Ca5–Sb6	3.553(1)
Eu/Ca2–Sb1	3.201(1)	Eu/Ca6–Sb1 × 2	3.3330(9)
Eu/Ca2–Sb2	3.236(1)	Eu/Ca6–Sb4 × 4	3.3623(7)
Eu/Ca2–Sb5 × 2	3.2649(8)	Cd1–Sb6	2.849(1)
Eu/Ca2–Sb3 × 2	3.3704(9)	Cd1–Sb3 × 2	2.8787(8)
Eu/Ca3–Sb2	3.230(1)	Cd1–Sb2	3.064(1)
Eu/Ca3–Sb1	3.280(1)	Cd2–Sb2 × 2	2.8085(7)
Eu/Ca3–Sb6 × 2	3.4008(8)	Cd2–Sb4	2.866(1)
Eu/Ca3–Sb4 × 2	3.4503(9)	Cd2–Sb5	2.978(1)
Eu/Ca4–Sb1	3.348(1)	Cd3–Sb1 × 2	2.7659(7)
Eu/Ca4–Sb3	3.369(1)	Cd3–Sb4	2.934(1)
Eu/Ca4–Sb3 × 2	3.3791(9)	Cd3–Sb5	3.193(1)
Eu/Ca4–Sb6 × 2	3.5313(9)	Sb5–Sb5	2.814(2)

## Supplementary Materials

The following are available online at http://www.mdpi.com/1996-1944/11/11/2146/s1, Table S1: Atomic coordinates and equivalent displacement parameters for Ca_1−*x*_Eu*_x_*Cd_2_Sb_2_ (*x* = 0.547(4)), Table S2: Atomic coordinates and equivalent displacement parameters for Ca_1−*x*_Eu*_x_*Cd_2_Sb_2_ (*x* = 0.731(4)), Table S3: Atomic coordinates and equivalent displacement parameters for Ca_1−*x*_Eu*_x_*Cd_2_Sb_2_ (*x* = 0.855(5)), Table S4: Atomic coordinates and equivalent displacement parameters for Ca_1−*x*_Eu*_x_*Cd_2_Sb_2_ (*x* = 0.924(4)), Table S5: Atomic coordinates and equivalent displacement parameters for EuCd_2_Sb_2_. Figure S1. Experimental and simulated powder diffraction patterns of Ca_1−*x*_Eu*_x_*Cd_2_Sb_2_ (*x* = 0.924(4)).

## References

[1] Kauzlarich S.M., Brown S.R., Snyder G.J. (2007). Zintl Phases for Thermoelectric Devices. Dalton Trans..

[2] Xia S., Bobev S. (2007). Diverse Polyanions Based on MnBi_4_ and MnSb_4_ Tetrahedra: Polymorphism, Structure, and Bonding in Ca_21_Mn_4_Bi_18_ and Ca_21_Mn_4_Sb_18_. Inorg. Chem..

[3] Xia S.-Q., Bobev S. (2008). Zintl Phase Variations through Cation Selection. Synthesis and Structure of *A*_21_Cd_4_*Pn*_18_ (*A* = Eu, Sr, Ba; *Pn* = Sb, Bi). Inorg. Chem..

[4] He H., Tyson C., Bobev S. (2011). New Compounds with [As_7_]^3−^ Clusters: Synthesis and Crystal Structures of the Zintl Phases Cs_2_NaAs_7_, Cs_4_ZnAs_14_ and Cs_4_CdAs_14_. Crystals.

[5] He H., Tyson C., Saito M., Bobev S. (2012). Synthesis and Structural Characterization of the Ternary Zintl Phases *AE*_3_Al_2_*Pn*_4_ and *AE*_3_Ga_2_*Pn*_4_ (*AE* = Ca, Sr, Ba, Eu; *Pn* = P, As). J. Solid State Chem..

[6] Wang Y., Darone G.M., Bobev S. (2016). The New Zintl Phases Eu_21_Cd_4_Sb_18_ and Eu_21_Mn_4_Sb_18_. J. Solid State Chem..

[7] Ovchinnikov A., Bobev S. (2018). Zintl Phases with Group 15 Elements and the Transition Metals: A Brief Overview of Pnictides with Diverse and Complex Structures. J. Solid State Chem..

[8] Nesper R. (2014). The Zintl-Klemm Concept—A Historical Survey. Z. Anorg. Allg. Chem..

[9] Brown S.R., Kauzlarich S.M., Gascoin F., Snyder G.J. (2006). Yb_14_MnSb_11_: New High Efficiency Thermoelectric Material for Power Generation. Chem. Mater..

[10] Aydemir U., Zevalkink A., Ormeci A., Wang H., Ohno S., Bux S., Snyder G.J. (2015). Thermoelectric Properties of the Zintl Phases Yb_5_*M*_2_Sb_6_ (*M* = Al, Ga, In). Dalton Trans..

[11] Ohno S., Aydemir U., Amsler M., Pöhls J.-H., Chanakian S., Zevalkink A., White M.A., Bux S., Wolverton C., Snyder G.J. (2017). Achieving zT > 1 in Inexpensive Zintl Phase Ca_9_Zn_4+*x*_Sb_9_ by Phase Boundary Mapping. Adv. Funct. Mater..

[12] Saparov B., Broda M., Ramanujachary K.V., Bobev S. (2010). New Quaternary Zintl Phases—Synthesis, Crystal and Electronic structures of K*A*_2_Cd_2_Sb_3_ (*A* = Ca, Sr, Ba, Eu, Yb). Polyhedron.

[13] Wang Y., Stoyko S., Bobev S. (2015). Quaternary Pnictides with Complex, Noncentrosymmetric Structures. Synthesis and Structural Characterization of the New Zintl Phases Na_11_Ca_2_Al_3_Sb_8_, Na_4_CaGaSb_3_, and Na_15_Ca_3_In_5_Sb_12_. Inorg. Chem..

[14] Devlin K.P., Kazem N., Zaikina J.V., Cooley J.A., Badger J.R., Fettinger J.C., Taufour V., Kauzlarich S.M. (2018). Eu_11_Zn_4_Sn_2_As_12_: A Ferromagnetic Zintl Semiconductor with a Layered Structure Featuring Extended Zn_4_As_6_ Sheets and Ethane-like Sn_2_As_6_ Units. Chem. Mater..

[15] (2014). SAINT.

[16] (2014). SADABS.

[17] Sheldrick G.M. (2015). Crystal Structure Refinement with SHELXL. Acta Crystallogr. Sect. C.

[18] Gelato L.M., Parthé E. (1987). STRUCTURE TIDY—A Computer Program to Standardize Crystal Structure Data. J. Appl. Cryst..

[19] Soler J.M., Artacho E., Gale J.D., García A., Junquera J., Ordejón P., Sánchez-Portal D. (2002). The SIESTA method for ab initio order-N materials simulation. J. Phys. Condens. Matter.

[20] Perdew J.P., Burke K., Ernzerhof M. (1996). Generalized Gradient Approximation Made Simple. Phys. Rev. Lett..

[21] Troullier N., Martins J.L. (1991). Efficient Pseudopotentials for Plane-wave Calculations. Phys. Rev. B.

[22] Dudarev S.L., Botton G.A., Savrasov S.Y., Humphreys C.J., Sutton A.P. (1998). Electron-energy-loss Spectra and the Structural Stability of Nickel Oxide: An LSDA+U Study. Phys. Rev. B.

[23] Flage-Larsen E., Diplas S., Prytz Ø., Toberer E.S., May A.F. (2010). Valence Band Study of Thermoelectric Zintl-phase SrZn_2_Sb_2_ and YbZn_2_Sb_2_: X-ray Photoelectron Spectroscopy and Density Functional Theory. Phys. Rev. B.

[24] Cooley J.A., Promkhan P., Gangopadhyay S., Donadio D., Pickett W.E., Ortiz B.R., Toberer E.S., Kauzlarich S.M. (2018). High Seebeck Coefficient and Unusually Low Thermal Conductivity Near Ambient Temperatures in Layered Compound Yb_2−*x*_Eu*_x_*CdSb_2_. Chem. Mater..

[25] Steinberg S., Dronskowski R. (2018). The Crystal Orbital Hamilton Population (COHP) Method as a Tool to Visualize and Analyze Chemical Bonding in Intermetallic Compounds. Crystals.

[26] Zhang H., Fang L., Tang M.-B., Chen H.-H., Yang X.-X., Guo X., Zhao J.-T., Grin Y. (2010). Synthesis and properties of CaCd_2_Sb_2_ and EuCd_2_Sb_2_. Intermetallics.

[27] Payne A.C., Sprauve A.E., Olmstead M.M., Kauzlarich S.M., Chan J.Y., Reisner B., Lynn J. (2002). Synthesis, Magnetic and Electronic Properties of Single Crystals of EuMn_2_P_2_. J. Solid State Chem..

[28] Bobev S., Merz J., Lima A., Fritsch V., Thompson J.D., Sarrao J.L., Gillessen M., Dronskowski R. (2006). Unusual Mn−Mn Spin Coupling in the Polar Intermetallic Compounds CaMn_2_Sb_2_ and SrMn_2_Sb_2_. Inorg. Chem..

[29] Schellenberg I., Eul M., Hermes W., Pöttgen R. (2010). A ^121^Sb and ^151^Eu Mössbauer Spectroscopic Investigation of EuMn_2_Sb_2_, EuZn_2_Sb_2_, YbMn_2_Sb_2_, and YbZn_2_Sb_2_. Z. Anorg. Allg. Chem..

[30] Peng W., Chanakian S., Zevalkink A. (2018). Crystal Chemistry and Thermoelectric Transport of Layered *AM*_2×2_ compounds. Inorg. Chem. Front..

[31] Zhang H., Zhao J.-T., Grin Y., Wang X.-J., Tang M.-B., Man Z.-Y., Chen H.-H., Yang X.-X. (2008). A New Type of Thermoelectric Material, EuZn_2_Sb_2_. J. Chem. Phys..

[32] Wang X.-J., Tang M.-B., Chen H.-H., Yang X.-X., Zhao J.-T., Burkhardt U., Grin Y. (2009). Synthesis and High Thermoelectric Efficiency of Zintl Phase YbCd_2−*x*_Zn*_x_*Sb_2_. Appl. Phys. Lett..

[33] Cao Q.-G., Zhang H., Tang M.-B., Chen H.-H., Yang X.-X., Grin Y., Zhao J.-T. (2010). Zintl Phase Yb_1−*x*_Ca*_x_*Cd_2_Sb_2_ with Tunable Thermoelectric Properties Induced by Cation Substitution. J. Appl. Phys..

[34] Zhang H., Baitinger M., Tang M.-B., Man Z.-Y., Chen H.-H., Yang X.-X., Liu Y., Chen L., Grin Y., Zhao J.-T. (2010). Thermoelectric Properties of Eu(Zn_1−*x*_Cd*_x_*)_2_Sb_2_. Dalton Trans..

[35] Zhang H., Fang L., Tang M.-B., Man Z.Y., Chen H.H., Yang X.X., Baitinger M., Grin Y., Zhao J.-T. (2010). Thermoelectric Properties of Yb*_x_*Eu_1−*x*_Cd_2_Sb_2_. J. Chem. Phys..

[36] Guo K., Cao Q.-G., Feng X.-J., Tang M.-B., Chen H.-H., Guo X., Chen L., Grin Y., Zhao J.-T. (2011). Enhanced Thermoelectric Figure of Merit of Zintl Phase YbCd_2−*x*_Mn*_x_*Sb_2_ by Chemical Substitution. Eur. J. Inorg. Chem..

[37] Shannon R.D. (1976). Revised Effective Ionic Radii and Systematic Studies of Interatomic Distances in Halides and Chalcogenides. Acta Crystallogr. Sect. A.

[38] Xia S., Bobev S. (2007). Cation−Anion Interactions as Structure Directing Factors: Structure and Bonding of Ca_2_CdSb_2_ and Yb_2_CdSb_2_. J. Am. Chem. Soc..

[39] Saparov B., Saito M., Bobev S. (2011). Syntheses, and Crystal and Electronic Structures of the New Zintl Phases Na_2_*A*CdSb_2_ and K_2_*A*CdSb_2_ (*A* = Ca, Sr, Ba, Eu, Yb): Structural Relationship with Yb_2_CdSb_2_ and the Solid Solutions Sr_2−*x*_*A_x_*CdSb_2_, Ba_2−*x*_*A_x_*CdSb_2_ and Eu_2−*x*_Yb*_x_*CdSb_2_. J. Solid State Chem..

[40] Wang J., Yang M., Pan M.-Y., Xia S.-Q., Tao X.-T., He H., Darone G., Bobev S. (2011). Synthesis, Crystal and Electronic Structures, and Properties of the New Pnictide Semiconductors *A*_2_Cd*Pn*_2_ (*A* = Ca, Sr, Ba, Eu; *Pn* = P, As). Inorg. Chem..

[41] Wilson D., Saparov B., Bobev S. (2011). Synthesis, Crystal Structures and Properties of the Zintl phases Sr_2_ZnP_2_, Sr_2_ZnAs_2_, *A*_2_ZnSb_2_ and *A*_2_ZnBi_2_ (*A* = Sr and Eu). Z. Anorg. Allg. Chem..

[42] Park S.-M., Kim S.-J., Kanatzidis M.G. (2005). Sr_2_MnSb_2_: A New Ternary Transition Metal Zintl Phase. Inorg. Chem..

[43] Ovchinnikov A., Saparov B., Xia S.-Q., Bobev S. (2017). The Ternary Alkaline-Earth Metal Manganese Bismuthides Sr_2_MnBi_2_ and Ba_2_Mn_1−*x*_Bi_2_ (*x* ≈ 0.15). Inorg. Chem..

[44] Ovchinnikov A., Makongo J.P.A., Bobev S. (2018). Yet Again, New Compounds Found in Systems with Known Binary Phase Diagrams. Synthesis, Crystal and Electronic Structure of Nd_3_Bi_7_ and Sm_3_Bi_7_. Chem. Commun..

[45] Park S.-M., Kim S.-J. (2004). Sr_11_Cd_6_Sb_12_: A New Zintl Compound with Infinite Chains of Pentagonal Tubes. J. Solid State Chem..

[46] Saparov B., Bobev S., Ozbay A., Nowak E.R. (2008). Synthesis, Structure and Physical Properties of the New Zintl Phases Eu_11_Zn_6_Sb_12_ and Eu_11_Cd_6_Sb_12_. J. Solid State Chem..

[47] Kazem N., Cooley J., Burks E.C., Liu K., Kauzlarich S.M. (2016). Synthesis, Characterization, and Low Temperature Transport Properties of Eu_11–*x*_Yb*_x_*Cd_6_Sb_12_ Solid-Solution Zintl Phases. Inorg. Chem..

[48] Xia S.-Q., Bobev S. (2008). Are Ba_11_Cd_6_Sb_12_ and Sr_11_Cd_6_Sb_12_ Zintl Phases or Not? A Density-functional Theory Study. J. Comp. Chem..

[49] Saparov B., Bobev S. (2010). Undeca-europium Hexa-zinc Dodeca-arsenide. Acta Crystallogr. Sect. E Struct. Rep. Online.

