# Supplementary material for: Exploratory Work in the Quaternary System of Ca–Eu–Cd–Sb: Synthesis, Crystal, and Electronic Structures of New Zintl Solid Solutions
Source: Materials (Basel). 2018 Oct 31;11(11):2146. doi: 10.3390/ma11112146 (PMC6265713; doi:10.3390/ma11112146)
Supplement: Supplementary file 1 [file materials-11-02146-s001.pdf]

materials

Exploratory work in the quaternary system Ca–Eu–Cd–Sb: Synthesis, crystal and electronic structures of new Zintl solid solutions

Alexander Ovchinnikov [1], Gregory M. Darone [1,2], Bayrammurad Saparov [1,3] and Svilen Bobev [1,*]

[1] Department of Chemistry and Biochemistry, University of Delaware, Newark, DE 19716, USA
[2] Charter School of Wilmington, Wilmington, DE 19807, USA
[3] Department of Chemistry and Biochemistry, University of Oklahoma, Norman, OK 73019, USA
* Correspondence: bobev@udel.edu; Tel.: +1-302-831-8720

Supporting information

Table S1. Atomic coordinates and equivalent displacement parameters for $Ca_{1-x}Eu_xCd_2Sb_2$, x = 0.547(4).

Atom	Site	*x*	*y*	*z*	U_{eq} [a]
Ca/Eu [b]	1*a*	0	0	0	0.0127(3)
Cd	2*d*	1/3	2/3	0.36908(6)	0.0152(2)
Sb	2*d*	1/3	2/3	0.75658(5)	0.0122(2)

[a] U_{eq} is defined as one third of the trace of the orthogonalized U_{ij} tensor. [b] 0.452Ca+0.548(4)Eu.

Table S2. Atomic coordinates and equivalent displacement parameters for $Ca_{1-x}Eu_xCd_2Sb_2$, x = 0.731(4).

Atom	Site	*x*	*y*	*z*	U_{eq} [a]
Ca/Eu [b]	1*a*	0	0	0	0.0126(3)
Cd	2*d*	1/3	2/3	0.36857(8)	0.0148(2)
Sb	2*d*	1/3	2/3	0.75500(7)	0.0120(2)

[a] U_{eq} is defined as one third of the trace of the orthogonalized U_{ij} tensor. [b] 0.269Ca+0.731(4)Eu.

Table S3. Atomic coordinates and equivalent displacement parameters for $Ca_{1-x}Eu_xCd_2Sb_2$, x = 0.855(5).

Atom	Site	*x*	*y*	*z*	U_{eq} [a]
Ca/Eu [b]	1*a*	0	0	0	0.0108(3)
Cd	2*d*	1/3	2/3	0.3684(1)	0.0134(2)
Sb	2*d*	1/3	2/3	0.75384(9)	0.0106(2)

[a] U_{eq} is defined as one third of the trace of the orthogonalized U_{ij} tensor. [b] 0.145Ca+0.855(5)Eu.

Table S4. Atomic coordinates and equivalent displacement parameters for $Ca_{1-x}Eu_xCd_2Sb_2$, x = 0.924(4).

Atom	Site	*x*	*y*	*z*	U_{eq} [a]
Ca/Eu [b]	1*a*	0	0	0	0.0108(2)
Cd	2*d*	1/3	2/3	0.36807(8)	0.0129(2)
Sb	2*d*	1/3	2/3	0.75340(6)	0.0096(2)

[a] U_{eq} is defined as one third of the trace of the orthogonalized U_{ij} tensor. [b] 0.076Ca+0.924(4)Eu.

Table S5. Atomic coordinates and equivalent displacement parameters for $EuCd_2Sb_2$.

Atom	Site	*x*	*y*	*z*	U_{eq} [a]
Eu	1*a*	0	0	0	0.0139(2)
Cd	2*d*	1/3	2/3	0.36796(8)	0.0146(2)
Sb	2*d*	1/3	2/3	0.75321(7)	0.0114(2)

[a] U_{eq} is defined as one third of the trace of the orthogonalized U_{ij} tensor.

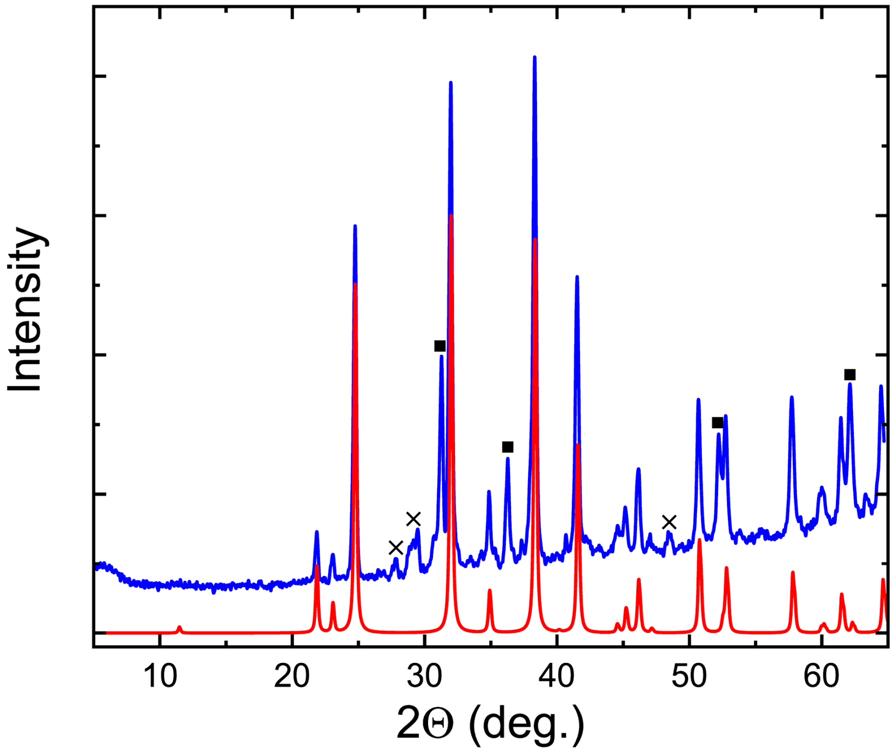

Figure S1. Powder diffraction pattern of a representative $Ca_{1-x}Eu_xCd_2Sb_2$ sample. Experimental and simulated from single crystal diffraction data plots are shown in blue and red, respectively (data used in the calculation were from the refinement of $Ca_{1-x}Eu_xCd_2Sb_2$, x = 0.924(4). Black squares and crosses indicate the Bragg peak positions of the remaining Pb flux and an unidentified impurity, respectively.
